# Histone acetyltransferase TGF-1 regulates *Trichoderma atroviride* secondary metabolism and mycoparasitism

**DOI:** 10.1371/journal.pone.0193872

**Published:** 2018-04-30

**Authors:** Elida Yazmín Gómez-Rodríguez, Edith Elena Uresti-Rivera, Olga Araceli Patrón-Soberano, María Auxiliadora Islas-Osuna, Alberto Flores-Martínez, Lina Riego-Ruiz, María Teresa Rosales-Saavedra, Sergio Casas-Flores

**Affiliations:** 1 División de Biología Molecular, IPICYT, San Luis Potosí, San Luis Potosí, Mexico; 2 Laboratorio de Genética y Biología Molecular de Plantas, Centro de Investigación en Alimentación y Desarrollo, Hermosillo, Sonora, Mexico; 3 Departamento de Biología, División de Ciencias Naturales y Exactas, Universidad de Guanajuato, Guanajuato, Guanajuato, Mexico; Universita degli Studi di Pisa, ITALY

## Abstract

Some filamentous fungi of the *Trichoderma* genus are used as biocontrol agents against airborne and soilborne phytopathogens. The proposed mechanism by which *Trichoderma* spp. antagonizes phytopathogens is through the release of lytic enzymes, antimicrobial compounds, mycoparasitism, and the induction of systemic disease-resistance in plants. Here we analyzed the role of TGF-1 (Trichoderma Gcn Five-1), a histone acetyltransferase of *Trichoderma atroviride*, in mycoparasitism and antibiosis against the phytopathogen *Rhizoctonia solani*. Trichostatin A (TSA), a histone deacetylase inhibitor that promotes histone acetylation, slightly affected *T*. *atroviride* and *R*. *solani* growth, but not the growth of the mycoparasite over *R*. *solani*. Application of TSA to the liquid medium induced synthesis of antimicrobial compounds. Expression analysis of the mycoparasitism-related genes *ech-42* and *prb-1*, which encode an endochitinase and a proteinase, as well as the secondary metabolism-related genes *pbs-1* and *tps-1*, which encode a peptaibol synthetase and a terpene synthase, respectively, showed that they were regulated by TSA. A *T*. *atroviride* strain harboring a deletion of *tgf-1* gene showed slow growth, thinner and less branched hyphae than the wild-type strain, whereas its ability to coil around the *R*. *solani* hyphae was not affected. Δ*tgf-1* presented a diminished capacity to grow over *R*. *solani*, but the ability of its mycelium -free culture filtrates (MFCF) to inhibit the phytopathogen growth was enhanced. Intriguingly, addition of TSA to the culture medium reverted the enhanced inhibition growth of Δ*tgf-1* MFCF on *R*. *solani* at levels compared to the wild-type MFCF grown in medium amended with TSA. The presence of *R*. *solani* mycelium in the culture medium induced similar proteinase activity in a Δ*tgf-1* compared to the wild-type, whereas the chitinolytic activity was higher in a Δ*tgf-1* mutant in the absence of *R*. *solani*, compared to the parental strain. Expression of mycoparasitism- and secondary metabolism-related genes in Δ*tgf-1* was differentially regulated in the presence or absence of *R*. *solani*. These results indicate that histone acetylation may play important roles in the biocontrol mechanisms of *T*. *atroviride*.

## Introduction

Nucleosome, the basic unit of chromatin, consists of ~146 base pairs (bp) of DNA wrapped around a histone octamer composed of two copies of each of the core histones H2A, H2B, H3, and H4 [1]. In eukaryotes, gene transcription is strongly dependent on chromatin compactness. Relaxed chromatin (euchromatin) comprises most of the active genes, whereas tightly packed chromatin (heterochromatin) comprehends most of the repressed genes [2,3]. Chemical modifications of histone N-terminal tails, including phosphorylation, methylation, ADP-ribosylation, and acetylation, among others, strongly correlate with chromatin structure and gene regulation [3]. Among all known histone tails’ chemical modifications, probably histone acetylation is the best understood to date. Histone acetylation levels result from the balance of the activities of histone acetyltransferases (HATs) and histone deacetylases (HDACs). Acetylation of lysine residues in histone N-terminal tails is related to a relaxed chromatin leading to gene transcription, whereas deacetylation is tightly associated with heterochromatin resulting in gene repression [4]. Several studies have revealed important roles of histone acetylation in gene transcription of various cellular processes [4]. However, some reports have shown that HATs and HDACs are also required for gene repression and activation, respectively [5–7]. Histone acetylation is achieved by larger multisubunit complexes that are recruited by specific gene promoters to modify local chromatin structure and, thus, regulate transcription [8]. The HAT Gcn5 (general control nonderepressible-5) is part of the SAGA complex (Spt/Ada/Gcn5 L-acetyltransferase) and is the most studied member of the GNAT family (N-acetyltransferase family related to Gcn5), which preferentially acetylates lysines 9, 14, 18, and 27 of histone H3 [9]. In the budding yeast *Saccharomyces cerevisiae*, histone acetylation has been widely studied; however, in filamentous fungi this regulation mechanism has been poorly approached. Orthologous proteins to Gcn5 have been identified in filamentous fungi [5, 10–14]. In *Neurospora crassa*, the orthologous to Gcn5, NGF-1 acetylates lysine 14 of histone H3 through its association with the photoreceptor White Collar-1 for light activation of the *al-3* promoter [5, 15]. Recently, the participation of histone acetylation in development, as well as in primary and secondary metabolism through the HDAC HdaA, and the orthologous to Gcn5, GcnE has been described in *Aspergilli* [11, 16–18]. In *Ustilago maydis*, a maize pathogen, Gcn5 plays important roles in gene regulation, affecting virulence and dimorphic transition of yeast to mycelium [13, 14]. Furthermore, in *Trichoderma reesei*, TrGcn5 regulates mycelial growth, conidiation, and cellulase gene expression [10].

The *Trichoderma* genus comprises a group of cosmopolitan microorganisms with a variety of lifestyles and high plasticity to adapt to different ecosystems. For instance, *Trichoderma* spp. are commonly found growing in soil or as saprophytes in bark or decaying wood, as well as in many other substrates [19]. Furthermore, it has been suggested that these fungi have, as an ancestral lifestyle, the ability to antagonize other fungi by means of mycoparasitism [20]. This is one of the many reasons why they are used in biological control of soil- and air-borne phytopathogenic fungi [21]. In addition to the mycophagy behavior of *Trichoderma* to antagonize phytopathogenic microorganisms, other mechanisms such as competition for space and nutrients, antibiosis, and the activation of plant systemic disease-resistance have been proposed [22–25]. Mycoparasitism initiates by the recognition and adhesion of *Trichoderma* to the host cell wall, followed by the hydrolysis of host hyphae by means of lytic enzymes, concluding with the uptake of the host cellular content [26]. The main known activities implicated in host cell wall degradation include chitinase, glucanase, N-acetylglucosaminidase, and protease activities [19, 27]. Integration of multiple copies of cell wall degrading enzymes or proteases encoding genes in the *Trichoderma* genome, has provided increased biocontrol activity. For instance, *Trichoderma* strains overexpressing either *ech-42*, encoding an endochitinase of the family 18 of the glycosyl hydrolases, or *prb-1*, which codes for a basic protease of the subtilisin type, provide more protection to plants against root and foliar pathogens [28–31].

Intriguingly, the cooperative effect between lytic enzymes and antibiotics to disrupt cell walls has been proposed [32]. In this sense, *Trichoderma* spp. use non-ribosomal peptide synthetases (NRPS) to produce linear peptides called peptaibols, which exert antibacterial, antifungal, and occasionally antiviral activities [33–35]. The peptaibol synthetase PBS-1 of *T*. *atroviride* contains 19 typical peptide synthetase modules with the required additional modifying domains at its N- and C-termini [36]. In fungi, the 4-phosphopantetheinyl transferase (PPTase) activates enzymes involved in primary and secondary metabolism [37]. In *T*. *virens*, a Δ*ppt1* mutant was unable to synthesize peptaibols, and was also incapable of inhibiting the growth of phytopathogenic fungi and oomycetes [38]. Moreover, in *T*. *virens*, a secondary metabolite deficient mutant showed a reduced expression of genes whose products are related to secondary metabolism. In agreement with these results, the fungus was incapable of synthetizing the antibiotics viridiol and viridin [39].

The search to identify regulators of mycoparasitism and secondary metabolism-related genes in the presence of external stimuli, such as the presence of a host, needs to consider the way that leads to chromatin modifications in *Trichoderma*.

In this work, we analyzed the effect of Trichostatine A (TSA), an inhibitor of HDACs from class I and II [40], in mycoparasitism, in the production of antibiotics, and in the expression of mycoparasitism- and secondary metabolism-related genes in *T*. *atroviride* during its interaction with the phytopathogenic fungus *Rhizoctania solani*. The role of TGF-1, the orthologous to Gcn5 of *S*. *cerevisiae* in *T*. *atroviride*, on mycoparasitism and on the transcriptional regulation of mycoparasitism and secondary metabolism-related genes was also assessed. Our results provide new insights into the biological control mechanisms of phytopathogens by *Trichoderma*.

## Materials and methods

### Microorganisms and growth conditions

*T*. *virens* Gv29-8 [41], *T*. *atroviride* IMI206040 and Δ*tgf*-1 (Uresti-Rivera et al., in preparation) were used throughout this study. *Rhizoctonia solani*, *Trichoderma citrinoviride* and *Trichoderma harzianum* strains were isolated from a tomato field in San Luis Potosi, Mexico (22° 38' 39.84" N, 100° 50' 56.4" W), and identified by PCR amplification of the internal transcribed spacer (ITS) of ribosomal deoxyribonucleic acid (rDNA) sequences, using the oligonucleotides ITS1 and ITS4 [42]. Fungal strains were routinely grown in potato dextrose agar (PDA) or potato dextrose broth (PDB) (both from Difco^™^, BD Becton, Dickinson and Company, New Jersey, USA), as indicated for each experiment. When indicated, 300 nM of TSA (Sigma-Aldrich, Taufkirchen, Germany) was added to the media. All the strains were incubated at 28 °C at the indicated times for each experiment.

### Dual cultures of *T*. *atroviride* wild-type and Δ*tgf-1* strains *versus R*. *solani* and other *Trichoderma* species

A plug of actively growing mycelium of *T*. *atroviride* wild-type (wt) strain was placed at one edge of the Petri dish, whereas at the opposite edge a plug of actively growing mycelium of *R*. *solani*, *T*. *virens*, *T*. *citrinoviride* or *T*. *harzianum* was placed. The dual cultures were incubated at 28 °C for 36, 48, 60, 72, 96, 120, 168, and 196 h and photographed. Radial growth inhibition of *R*. *solani* by *T*. *atroviride* strains was determined after 36, 48, 60, 72 and 96 h post-inoculation. The Δ*tgf-1* inocula were obtained by growing the fungus at 28 °C for 96 h in PDB at 250 rpm. The mycelium was centrifuged 5 min at 2000 rpm and used to inoculate PDA plates at 3 cm from the middle, and allowed to grow for 72 h before inoculating with the other fungal strains, as described above. Two independent experiments were performed in triplicate. Photographs were taken at the indicated times. In addition, dual cultures of *T*. *atroviride* wt or Δ*tgf-1* against *R*. *solani* were carried out on PDA plates with or without TSA (300 nM). The different fungal strains were grown alone on PDA plates with or without TSA as controls of the interactions.

### Antimicrobial activity of *T*. *atroviride* wt and Δ*tgf-1* mycelium-free culture filtrates *versus R*. *solani*

*T*. *atroviride* wt and Δ*tgf-1* strains were grown in PDB or Vogel minimal medium supplemented with 1.5% sucrose [43] in the presence or absence of 300 nM TSA for 7 days. Then, cultures were filtered using Corning^®^ 500 ml bottle top vacuum filter, 0.2 μm-pore 33.2 cm^2^ nylon membrane (Corning Life Sciences, Massachusetts, USA). Mycelium-free culture filtrates (MFCF) were used to prepare 1 × PDA plates containing 60% of MFCF and 40% of sterile distilled water. A mycelial plug of an actively growing colony of *R*. *solani* was inoculated on the center of a PDA-MFCF plate, incubated at 28 °C, and radial growth was measured after 12, 24, 36, 48, and 60 h post-inoculation. PDA plates without MFCF inoculated with *R*. *solani* were included as controls. Two independent experiments were performed in triplicate.

### Induction of *Trichoderma* cultures with *R*. *solani* mycelium

*R*. *solani* mycelium was grown in PDB medium for 7 days and filtered as described for MFCF. After filtration, *R*. *solani* mycelium was lyophilized, frozen in liquid nitrogen, and ground. For the induction of *T*. *atroviride* wt or Δ*tgf-1*, 0.7 g of *R*. *solani* sterilized powder was added to 500 ml of PDB previously autoclaved. The induction medium was inoculated with 10 plugs (0.5 cm^2^) of *T*. *atroviride* wt or Δ*tgf-1* strains and incubated at 28 °C with agitation at 250 rpm for 7 days in the darkness. The wt and Δ*tgf-1* induced MFCF were obtained by filtration as described above for *R*. *solani*. The mycelium was discarded and the MFCF were concentrated by lyophilization. The wt and Δ*tgf-1* lyophilized samples were resuspended in 500 μl of phosphate buffer, and protein concentration was determined as described by [44].

### Relative expression of mycoparasitism (*ech-42* and *prb-1*) and secondary metabolism-related genes (*tps-1* and *pbs-1*) in dual culture experiments

Dual cultures of *T*. *atroviride* wt and Δ*tgf-1* against *R*. *solani* were performed as described above, but placing a sterile cellophane sheet on the PDA plates before inoculation of the fungi. Dual cultures were incubated for 36, 48, and 60 h at 28 °C. Mycelia of the different *Trichoderma* strains were collected at the indicated times, frozen in liquid nitrogen, and ground for total RNA extraction by the Trizol^®^ method (Invitrogen, New York, USA). Total RNA (5 μg) was treated with Turbo DNase as described by the manufacturer (Ambion, Life Technologies, New York, USA). cDNA was synthesized using the Superscript^®^ kit (Invitrogen), following the manufacturer’s protocol. Subsequently, cDNA was quantified using a Nanodrop spectrophotometer (ND-1000, Thermo Scientific, Delaware, USA). Relative gene expression was assessed by quantitative reverse transcription PCR (RT-qPCR), using specific oligonucleotides (Table 1) in an Applied Biosystems 7500/7500 Fast Real-Time PCR System, using the Fast SYBR^®^ Green Master Mix (Applied Biosystems, Foster City, California), in a final volume of 20 μl per reaction. Reactions were set up by using the standard settings of the system, except for the oligonucleotides annealing temperature (Table 1), which was 62 °C. Two independent experiments were performed in triplicate. Relative genes expression was calculated using the 2^(ΔΔ-Ct)^ method [45].

**Table 1:** Oligonucleotide used in this study.

Gene name	*JGI Protein ID	Primer sequence (5’ to 3’)	Primer name	Annealing temperature	Product size (bp)
***ech-42***	176466	atgttgggcttcctcggaaaatcc	Ech-42 fw	60 °C	237
		ccaggttctgaggctggaagtt	Ech-42 Rv		
***prb-1***	268415	atgaccagcattcgtcgtctcgct	Prb-1 fw	60 °C	206
		gcagtgctacgcttggtcaacga	Prb-1 Rv		
***pbs-1***	317938	ccgagacaagcgtcaagga	Pbs-1 fw	60 °C	152
		cgctctgcgcattggtt	Pbs-1 Rv		
***tps-1***	31441	ccatgttgagctccttcttcaa	Tps-1 fw	60 °C	150
		cgacggtgacttgcttaacg	Tps-1 Rv		
***act-1***	297070	tcaccgaggcccccatcaacc	Act fw	60 °C	127
		cgaccggaagcgtacagggacaga	Act Rv		

*JGI, Joint Genome Institute

### Scanning Electron Microscopy (SEM) micrographs of *T*. *atroviride* wt and Δ*tgf-1 versus R*. *solani*

For SEM analysis, dual cultures of *T*. *atroviride* wt and Δ*tgf-1* strains against themselves or against *R*. *solani* were performed as described in the previous section. For SEM analysis, 0.5-cm^2^ agar pieces of the different confrontations at the indicated times were fixed in 3% glutaraldehyde in PBS (phosphate buffered saline) for 2 h. Samples were rinsed three times with cold PBS for 15 min each. Afterwards, samples were post-fixed with 1% OsO_4_ in PBS solution for 1 h, followed by three rinses in PBS, 15 min each, and then dehydrated in graded ethanol. Critical point drying was done in a Tousimis Samdri-PVT-3D, mounted, and gold-coated sputtering in the Cressington Model 108 auto, and examined in a FEI model Quanta 200 SEM. The SEM was adjusted at 18 kV, spot 5, WD 10 mm, and the photomicrographs were taken with Everhart Thornley Detector (ETD).

### Proteinase activity

Proteinase activity was determined in a 500-μl reaction mix containing 100 μl of *Trichoderma atroviride* wt and Δ*tgf-1* strains induced MFCF, 0.5 mM Suc-Ala-Ala-Pro-Phe-pNA (Sigma, Cat number S7388) in 50 mM MOPS, pH 7.0. The reaction mix was incubated at 37 °C for 20 min, and 500 μl of ice-cold water was added to stop the reaction, and measured spectrophotometrically at 405 nm. The activity was expressed as millimoles of p-nitroanilide released per minute [46]. Specific activity was referred to 1 mg of protein.

### Chitinolytic activity

Chitinolytic activity was assayed by estimating the reducing ends of sugars as described elsewhere [47]. The reactions were performed in a 600- μl mix containing 300 μl of 1% colloidal chitin, pH 6.6, and 300 μl of a 1/100 dilution of the wt or Δ*tgf-1* concentrated induced MFCF. The reaction mix was incubated 30 min at 37 °C, and the hydrolysis reactions were terminated and analyzed by adding 600 μl of dinitrosalicylic acid reagent (DNS). Then, the reaction mix was boiled for 15 min, and the insoluble chitin was removed by centrifugation, absorbance was measured spectrophotometrically at 540 nm. One unit of chitinase activity is defined as the amount of enzyme required to release 1 μmol of detectable reducing sugars in 1 min at 37 °C.

### Data analysis

Experiments were statistically analyzed in the SPSS 10 program (SPSS, Chicago). Multivariate analyzes with a Tukey’s post hoc test were used for testing differences in antimicrobial activity of MFCF, RT-qPCR and biochemical analysis (proteinase and chitinolytic activity). Linear regression analysis was done using the SPSS 10 program (SPSS, Chicago). Different letters are used to indicate means that differ significantly (P ≤ 0.05).

## Results

### Trichostatin A (TSA) did not affect the growth of *T*. *atroviride* over *R*. *solani*

**Fig 1:**
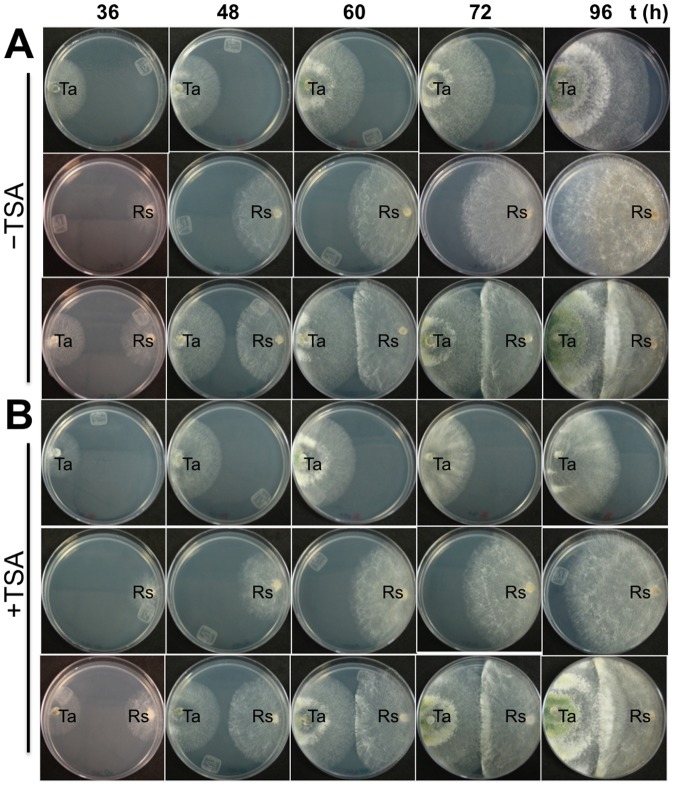
Trichostatin A did not affect the overgrowth of *Trichoderma atroviride* on *Rhizoctonia solani*. Dual confrontation plates of *T*. *atroviride* (left side) against *R*. *solani* (right side) on PDA plates in the absence (A) or presence (B) of 300 nM Trichostatin A, incubated at 28 °C and photographed at 36, 48, 60, 72, and 96 h. Ta = *T*. *atroviride*, Rs = *R*. *solani*, Trichostatin A = TSA. Images are representative of similar results from three independent trials.

**Fig 2:**
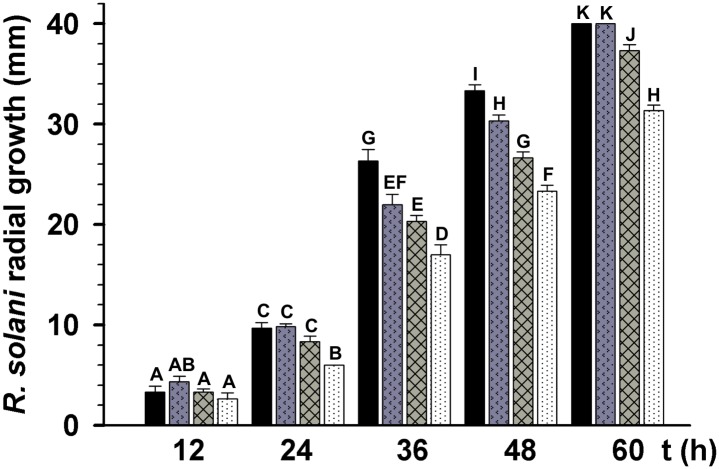
TSA enhances the antibiotic activity of *T*. *atroviride* mycelium-free culture filtrates (MFCF) against *R*. *solani*. *T*. *atroviride* was grown in PDB amended or not with 300 nM TSA, incubated for 7 days at 28 °C. The MFCFs were added to PDA 1× medium at a final concentration of 60%. *R*. *solani* radial growth was measured at 12, 24, 36, 48, and 60 h post-inoculation. *R*. *solani* growth on PDA (black bars), PDA plates amended with 300 nM TSA (arrow filled bars), PDA plates amended with 60% of *T*. *atroviride* MFCF (crosshatched bars), and PDA plates amended with 60% of *T*. *atroviride* MFCF grown in PDB plus TSA (black dotted bars) are shown. The bars show the mean ± SD of three independent biological replicates. Different letters are used to indicate means that differ significantly (*P <* 0.05). Three replicate plates were established for each treatment, and the experiment was repeated twice.

To elucidate the involvement of chromatin acetylation on the biocontrol capacities of *T*. *atroviride*, we first assessed the effect of the histone deacetylase (HDAC) inhibitor Trichostatin A (TSA) on the growth of *T*. *atroviride* over *R*. *solani* in dual cultures. *T*. *atroviride* was grown alone or in confrontation with *R*. *solani* on PDA plates amended or not with TSA (300 nM). Dual cultures were visually inspected and photographed after 36, 48, 60, 72, and 96 h of co-culture (Fig 1A and 1B). Addition of TSA to the growing medium slightly affected the growth of *T*. *atroviride* and *R*. *solani* strains (Figs 1B and 2) compared to the control without TSA (Figs 1A and 2). However, the capacity of *T*. *atroviride* to growth over *R*. *solani* was not affected by the presence (Fig 1B) or absence of TSA (Fig 1A).

### Addition of TSA to *T*. *atroviride*-growing medium enhanced its inhibitory effect on *R*. *solani* growth

As byproducts of their metabolism, most *Trichoderma* species are capable of synthetizing a wide range of antimicrobial compounds, used to combat phytopathogenic fungi. To determine whether TSA has an effect on the synthesis of antimicrobial compounds in *T*. *atroviride*, the fungus was grown for 7 days in PDB amended or not with 300 nM TSA. Thereafter, *Trichoderma* cultures were filtered to remove the mycelium. Plugs of *R*. *solani* were inoculated at the center of PDA plates amended with mycelium-free culture filtrates (MFCF) (final concentration of 60%) and the radial growth of *R*. *solani* was measured at 12, 24, 36, 48, and 60 h. As shown in Fig 2, addition of MFCF to PDA plates (crosshatched bars) inhibited *R*. *solani* radial growth compared to *R*. *solani* growth on PDA control plates with (arrow filled bars) or without TSA (black bars) (Fig 2). Furthermore, addition of MFCF plus 300 nM TSA to PDA plates (white dotted bars) enhanced the inhibition of *R*. *solani* radial growth compared to PDA-MFCF medium without TSA (crosshatched bars) (Fig 2).

### TSA and *R*. *solani* presence negatively affected *T*. *atroviride ech-42*, *prb-1*, *pbs-1*, and *tps-1* gene expression

**Fig 3:**
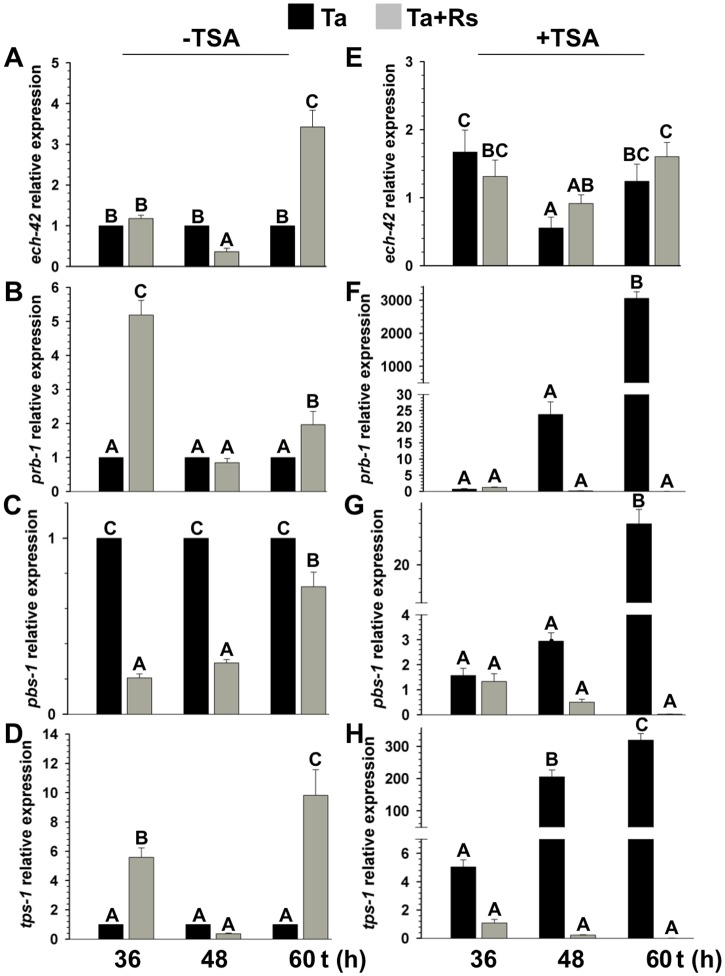
TSA and *R*. *solani* presence negatively affected *T*. *atroviride ech-42*, *prb-1*, *pbs-1*, and *tps-1* gene expression. *T*. *atroviride* and *R*. *solani* were co-cultured on PDA (A, B, C, and D) or PDA amended with 300 nM TSA (E, F, G, and H) at 28 °C. Total RNA was extracted from *T*. *atroviride* mycelium collected at 36, 48, and 60 h. Relative expression was calibrated using *act-1* as housekeeping gene and normalized against the wt strain in the absence of the phytopathogen. *ech-42* = 42-kDa endochitinase gene, *prb-1* = basic proteinase gene, *pbs-1* = peptaibol synthase gene, and *tps-1* = terpene synthase gene. Black bars, *T*. *atroviride* wt strain growing alone (Ta); grey bars, *T*. *atroviride* vs *R*. *solani* (Ta+Rs) co-cultures. The bars show the mean ± SD of three independent biological replicates. Different letters are used to indicate means that differ significantly (*P <* 0.05). This assay was repeated twice per triplicate with similar results.

To investigate whether the addition of TSA to the culture medium affected the expression of *T*. *atroviride* mycoparasitism-related genes *ech-42* and *prb-1*, and those related to secondary metabolism, *pbs-1* and *tps-1*, dual cultures of this fungus against *R*. *solani* were performed on PDA plates amended or not with 300 nM TSA. In the absence of TSA but in the presence of *R*. *solani*, expression of *ech-42* remained unalterable after 36 and 48 h of co-culture, whereas *prb-1* and *tps-1* were induced after 36 h. However, all three genes were repressed after 48 h and upregulated after 60 h of co-culture (grey bars) (Fig 3A, 3B and 3D). In contrast, *pbs-1* was downregulated at all tested times in the presence of the phytopathogen (grey bars) (Fig 3C). Addition of TSA to the culture medium in the absence of *R*. *solani* induced the expression of *ech-42*, and *tps-1* after 36 h (black bars); but *ech-42* was downregulated in the presence of *R*. *solani* (grey bars) (Fig 3E, 3G and 3H). Furthermore, expression of *tps-1* but no *prb-1*, *ech-42*, and *pbs-1* was considerably high at 48 h. However, *prb-1*, *tps-1*, and *pbs-1* expression was significantly high 60 h after the addition of TSA in the absence of *R*. *solani* (black bars), but downregulated or almost abolished in presence of the phytopathogen (grey bars), except *ech-42* (Fig 3F, 3G and 3H). The presence of TSA and the phytopathogen marginally downregulated the expression of *ech-42* after 36 and 48 h, which was marginally upregulated after 60 h of co-culture compared to their respective times in the presence of TSA alone (grey bars) (Fig 3E).

### Deletion of *tgf-1* did not affect *T*. *atroviride* mycoparasitic effect against *R*. *solani*

**Fig 4:**
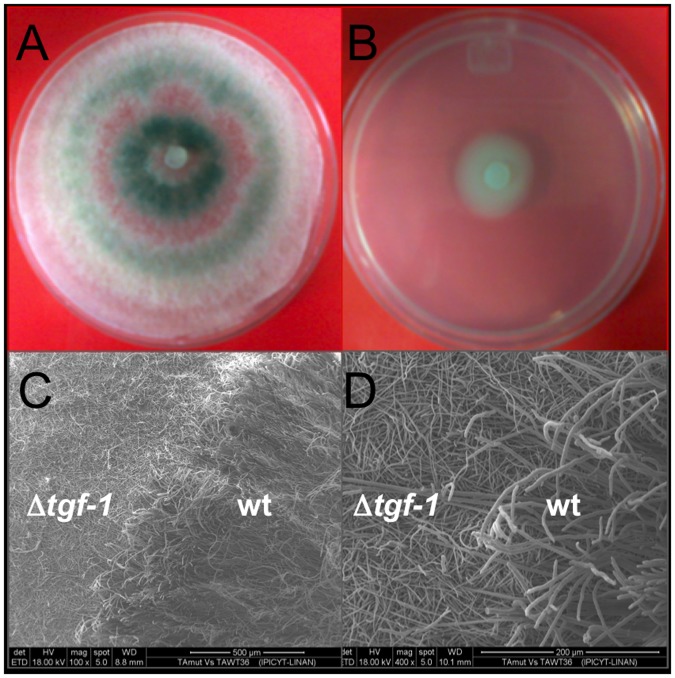
Colony morphology and development of *T*. *atroviride* wt and Δ*tgf-1* strains. *T*. *atroviride* wt (A) and Δ*tgf-1* (B) were grown on PDA plates for 7 days at 28 °C and photographed. Both strains were co-cultured on PDA medium, fixed, dehydrated, and analyzed by SEM at 100 (C) and 400 (D) magnifications. The wt is shown in the right side whereas the Δ*tgf-1* is shown in the left side of the photomicrographs. Images are representative of similar results from two independent trials, including three replicate plates.

The mutant growing phenotype of Δ*tgf-1*, whose wild-type gene encodes for a histone acetyltransferase, was analyzed by inoculating *T*. *atroviride* wt and Δ*tgf-1* on PDA plates during 72 h under 12:12 light:dark regime (Fig 4A and 4B). The Δ*tgf-1* strain showed a slow growth phenotype and lack of the typical green colored conidia compared to the wt strain (Fig 4A and 4B). SEM photomicrographs of the Δ*tgf-1* strain showed a thin, flat and less branched mycelium compared to the wt strain (Fig 4C and 4D).

**Fig 5:**
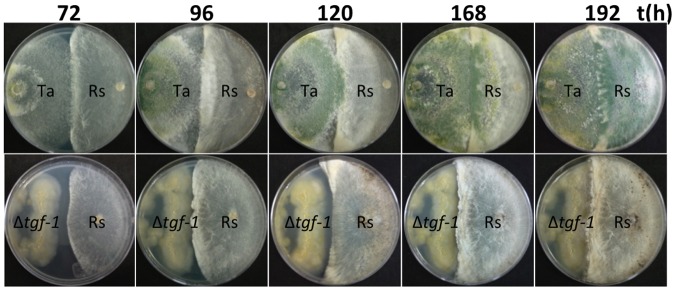
Dual culture assays of *T*. *atroviride* wt and Δ*tgf-1* strains *versus R*. *solani*. wt and Δ*tgf-1* strains were inoculated at the left side of the Petri dish, whereas *R*. *solani* was inoculated at the right side. Dual cultures were incubated at 28 °C, and photographed at 72, 96, 120, 168, and 192 h. Ta = *T*. *atroviride* wt strain, Δ*tgf-1 = T*. *atroviride tgf-1* mutant strain, Rs = *Rhizoctonia solani*. This assay was repeated twice including three replicate plates.

**Fig 6:**
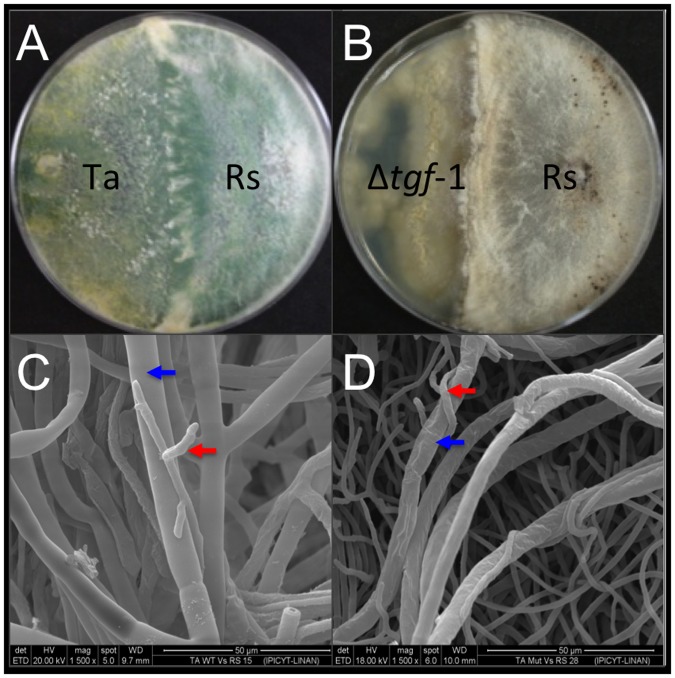
Deletion of *tgf-1* did not affect *T*. *atroviride* mycoparasitic effect against *R*. *solani*. Dual culture assays of *T*. *atroviride* wt (A) and Δ*tgf-1* (B) strains *versus R*. *solani* were analyzed by SEM (C and D, respectively). Blue arrowheads show *R*. *solani* hyphae, whereas red arrowheads show wt and Δ*tgf-1* coiling hyphae on *R*. *solani*. SEM photomicrographs were magnified 1500 ×. The experiments were repeated twice and representative photographs are shown.

To determine whether the product of *tgf-1* is involved in the antagonistic capacity of *T*. *atroviride* against phytopathogenic fungi, dual cultures of wt or Δ*tgf-1* strains against *R*. *solani* were conducted. The wt strain was able to growth over *R*. *solani*, whereas the Δ*tgf-1* strain was scarcely able to come in contact (Fig 5). Moreover, in confrontation assays of Δ*tgf-1* against the phytopathogen, the growth of the latter was slightly delayed as compared when *R*. *solani* was co-cultured with the wt strain (Fig 5). Linear regression analysis was used to find the relationship between the strains used in dual cultures and the growth inhibition of *R*. *solani* (S1 Table). Positive relationships were found between growth inhibition of *R*. *solani* by the wt, and growth inhibition of *R*. *solani* by the Δ*tgf-1* mutant. A strong significant positive (R^2^ = 0.802, Sig. = 0.000) relationship was observed in growth inhibition of *R*. *solani* by the wt, followed by a low, but significant positive relationship in growth inhibition of *R*. *solani* by Δ*tgf-1* mutant (R^2^ = 0.382, Sig. = 0.014). Since the Δ*tgf-1* mutant was barely able to enter in contact with the phytopathogen at the different times tested, the 192 h interaction zone of both dual cultures was taken and analyzed by SEM. Fig 6 shows that both, the Δ*tgf-1* and the wt, strains were able to coil around the phytopathogen hyphae (Fig 6C and 6D).

**Fig 7:**
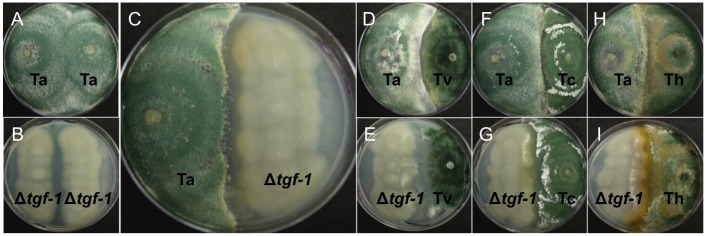
Antagonistic ability of Δ*tgf-1* strain against other *Trichoderma* species. Dual cultures of *T*. *atroviride* wt and Δ*tgf-1* strains against other species of *T*. *atroviride*, indicated on the right side of the figure, were grown at 28 °C for 96 h. Ta = *T*. *atroviride*, Δ*tgf-1* = *T*. *atroviride tgf-1* mutant strain, Tv = *T*. *virens*, Tc = *T*. *citrinoviride*, Th = *T*. *harzianum*. Images are representative of similar results from three independent trials.

Dual cultures of Δ*tgf-1* against itself (Fig 7B), its parental strain (Fig 7C), or other *Trichoderma* species (Fig 7D–7I) showed that Δ*tgf-1* could be overgrown by other *Trichoderma* species compared to its parental strain. Also, the Δ*tgf-1* mutant was able to stop the growth of other species, showing, in some cases, an enhanced brown color in the contact area, which is indicative of lysed hyphae (Fig 7G and 7I). Confrontation of *tgf-1* against itself did not show significant changes in any of the two colonies (Fig 7B); however, when the Δ*tgf-1* strain was confronted against its parental strain, a lysing zone was observed (Fig 7C).

### MFCF obtained from a Δ*tgf-1* strain shows enhanced inhibition of *R*. *solani* growth

**Fig 8:**
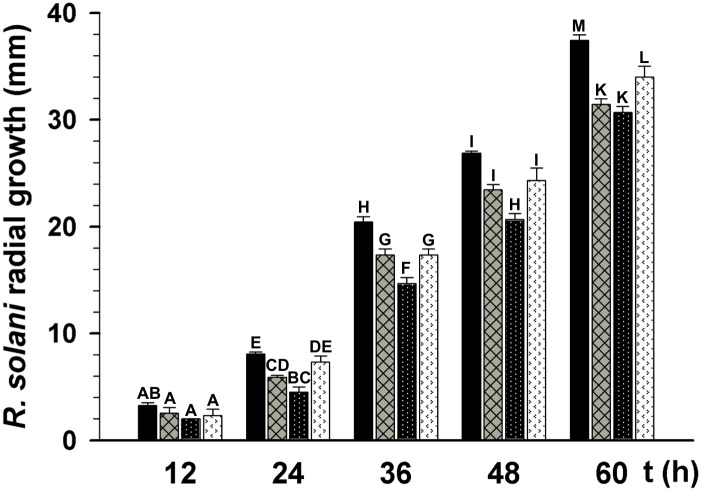
Antibiosis assay using MFCF obtained from *T*. *atroviride* wt or Δ*tgf-1* strains grown in the presence or absence of TSA against *R*. *solani*. The Δ*tgf-1* and wt strains were grown for 7 days in PDB medium amended or not with 300 nM TSA, at 28 °C. MFCFs obtained from each of these cultures were added to PDA 1× medium at a final concentration of 60%. *R*. *solani* was inoculated into the different media and its radial growth was determined at 12, 24, 36, 48, and 60 h. Radial growth of *R*. *solani* on PDA containing *T*. *atroviride* wt strain MFCF without TSA (black bars) or amended with TSA (crosshatched bars) was determined at the indicated times. Radial growth of *R*. *solani* on PDA containing *T*. *atroviride* Δ*tgf-1* strain MFCF without TSA (black dotted bars) or with TSA (arrow filled bars) was determined at the indicated times. The bars show the mean ± SD of three independent biological replicates. Different letters are used to indicate means that differ significantly (*P <* 0.05). Three replicate plates were established for each treatment, and the experiment was repeated twice.

Since the Δ*tgf-1* strain inhibited the growth of *R*. *solani* slightly more than the wt strain before they entered in contact (Fig 5 and S1 Table), we decided to assess the capacity of MFCF from Δ*tgf-1* to inhibit *R*. *solani* growth. PDA or PDA plus MFCF plates were inoculated in the center with mycelial plugs of *R*. *solani* and the radial growth of the phytopathogen was measured at 12, 24, 36, 48, and 60 h post-inoculation. MFCF from Δ*tgf-1* inhibited the growth of *R*. *solani* more effectively than that obtained from the wt strain (dotted black and black bars, respectively) (Fig 8). Intriguingly, addition of TSA to the Δ*tgf-1* growing medium diminished the negative effect of MFCF on the phytopathogen radial growth (arrow filled bars), compared to its respective control without TSA (dotted black bars), but MFCF from Δ*tgf-1* reached the inhibition effect observed with the wt MFCF plus TSA (cross-hatched bars) (Fig 8). Furthermore, the MFCF from the wt and Δ*tgf-1* grown in liquid Vogel’s minimal medium showed similar results compared when they were grown in PDB medium (S1 Fig and Fig 8, respectively). Addition of TSA to the wt, grown in Vogel’s minimal medium showed also similar results to those observed in PDB (Fig 8). Contrastingly, addition of TSA to the Vogel’s minimal medium increased the growth inhibition effect of the Δ*tgf-1* MFCF on *R*. *solani* compared when the mutant was grown in PDB plus TSA (S1 Fig).

### The Δ*tgf-1* strain shows constitutive chitinolytic but inductive proteolytic activity

**Fig 9:**
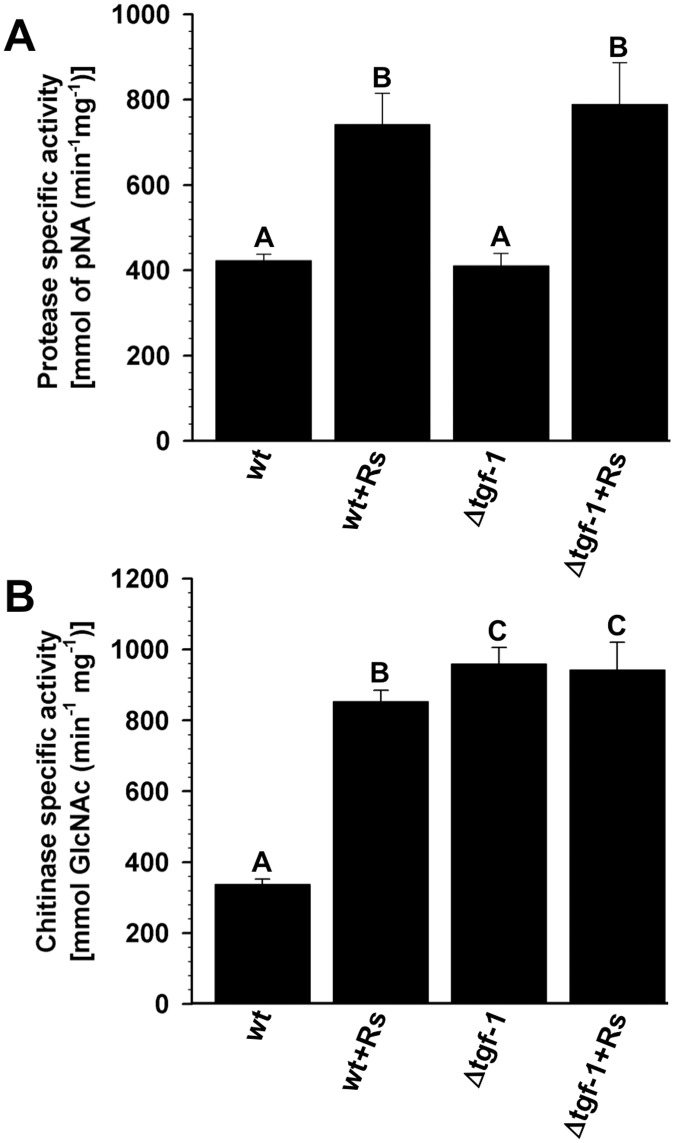
TGF-1 plays a negative role in chitinolytic activity but a minor role in proteolytic activity in *T*. *atroviride*. Chitinolytic activity was determined using colloidal chitin by estimating the reducing sugars with dinitro-salicylic acid (DNS) method, measuring absorbance spectrophotometrically at 540 nm. One unit of chitinase activity was defined as the amount of enzyme required to increase absorbance at 540 nm by 1 OD unit ml^-1^ h^-1^. Proteinase activity was determined using mM Suc-Ala-Ala-Pro-Phe-pNA (0.5 mM) as substrate, the liberation of pNA was measured spectrophotometrically at 405 nm. The activity was expressed as p-nitroanilide released per min. Specific activity was referred to 1 mg of protein. The effect of induced MFCF of the wt and Δ*tgf-1* strains was tested over both activities. The bars show the mean ± SD of three independent biological replicates. Different letters are used to indicate means that differ significantly (*P <* 0.05). Chitinolytic and proteolytic activities were tested in three replicates and repeated two times.

Lysing enzymes such as chitinases and proteases are used by some *Trichoderma* strains as biocontrol mechanisms against phytopathogenic fungi and oomycetes [23]. Aimed at elucidating whether chitinolytic and proteolytic activities were affected in the Δ*tgf-1* strain, and if they could be contributing to the enhanced inhibition of growth observed against *R*. *solani*, these activities were determined in cultures of the wt and Δ*tgf-1* strains induced by the presence of *R*. *solani* mycelium (Fig 9). Wild-type and Δ*tgf-1* MFCFs showed similar proteolytic activity under control conditions, whereas in the presence of *R*. *solani*, this activity increased at similar levels in both strains (Fig 9A). In contrast, the wt strain presented an increased chitinolytic activity in the presence of *R*. *solani*, compared to the mocked control, whereas the Δ*tgf-1* MFCF showed enhanced chitinolytic activity in control conditions, which was significantly high than that observed for the wt MFCF under the induced condition (Fig 9B). Addition of *R*. *solani* to the growing medium did not increase the chitinolytic activity of Δ*tgf-1* as compared to its own control (Fig 9B).

### TGF-1 and TSA differentially regulate *ech-42*, *prb-1*, *pbs-1*, and *tps-1* expression in the presence or absence of *R*. *solani*

**Fig 10:**
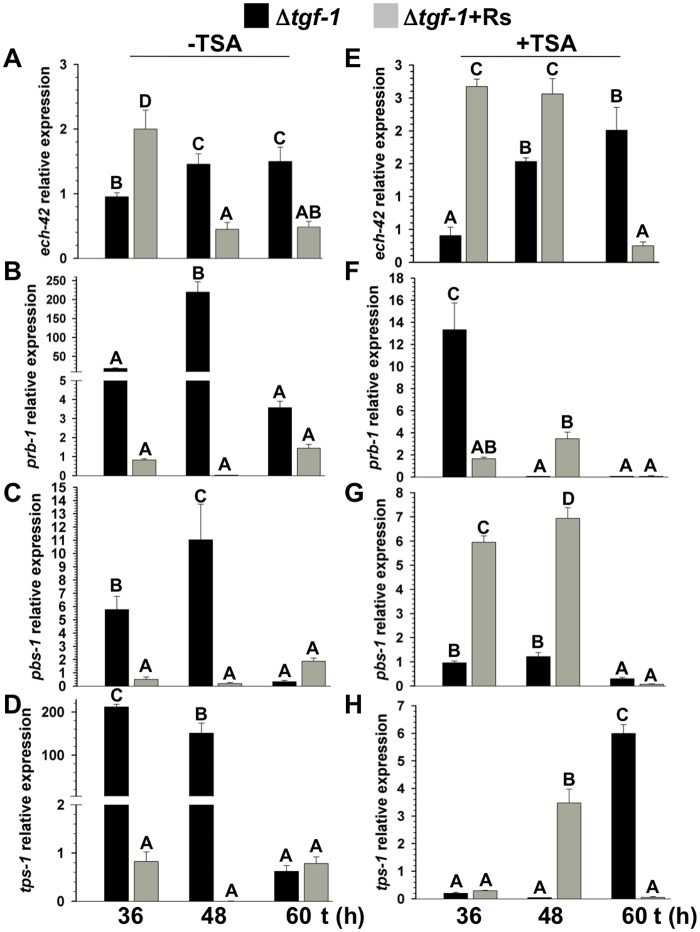
TGF-1 and TSA differentially regulate *T*. *atroviride ech-42*, *prb-1*, *pbs-1* and *tps*-1 in the presence or absence of *R*. *solani*. Dual culture assays of *T*. *atroviride* wt and Δ*tgf-1* against *R*. *solani*, or grown alone as control were performed on PDA in absence (A, B, C, D) or in presence of 300 nM TSA (E, F, G, H) at 28 °C. Total RNA was extracted from *T*. *atroviride* mycelium collected at 36, 48, and 60 h. Relative expression was calibrated using *act-1* as housekeeping gene and normalized against the wt strain in absence of the phytopathogen. *ech-42* = 42-kDa endochitinase gen, *prb-1* = basic proteinase gene, *pbs-1* = peptaibol synthase gene, *tps-1* = terpene synthase gene. Black bars represent Δ*tgf-1* strain, whereas gray bars represent Δ*tgf-1* vs *R*. *solani* interaction. Addition of TSA is indicated at the top of the panels. The bars show the mean ± SD of three independent biological replicates. Different letters are used to indicate means that differ significantly (*P <* 0.05). This assay was repeated twice per triplicate with similar results.

To investigate whether TGF-1 plays a role in the regulation of mycoparasitism- (*ech-42* and *prb-1*) and secondary metabolism-related genes (*pbs-1* and *tps-1*), their expression levels were analyzed in a Δ*tgf-1* background and compared to its parental strain during their co-culture with *R*. *solani*, in media amended or not with TSA (300 nM). In the absence of TSA and *R*. *solani*, *ech-42* showed significantly changes in its expression after 48 and 60 h (black bars) (Fig 10A), whereas *pbs-1*, and *tps-1* were considerably upregulated after 36 and 48 h, but downregulated at 60 h (black bars). *prb-1* was upregulated after 48 h but downregulated at 60 h (Fig 10B, 10C and 10D). The presence of *R*. *solani*, increased ~2-fold the expression of *ech-42* after 36 h of co-culture, but it was downregulated after 48 and 60 h (gray bars) (Fig 10A). *prb-1* expression suffered no significant changes in the presence of *R*. *solani* (Fig 10B). However, *pbs-1*, and *tps-1* were downregulated in the presence of the phytopathogen after 36 and 48 h compared to their controls in absence of *R*. *solani* (gray bars) (Fig 10B, 10C and 10D). In the presence of TSA, *ech-42* did not increase its expression after 36 h; however, it was marginally upregulated after 48 and 60 h (black bars) (Fig 10E). *pbs-1* was downregulated in the presence of TSA after 60 h, whereas *prb-1* and *tps-1* were repressed 48 and 60 h after the addition of TSA, respectively (black bars) (Fig 10E, 10F and 10G). The presence of both, the phytopathogen and TSA, upregulated the expression of *ech-42* and *pbs-1* after 36 and 48 h (gray bars) (Fig 10E and 10G). *prb-1* and *tps-1* were marginally upregulated in the presence of TSA and *R*. *solani* after 48 h, and downregulated after 60 h (gray bars) (Fig 10G and 10H).

## Discussion

Some species of the *Trichoderma* genus are used as biocontrol agents of phytopathogenic fungi and oomycetes that affect negatively important crops [22]. The molecular mechanisms governing mycoparasitism and secondary metabolism in *Trichoderma* have been intensely investigated. However, the role of histone acetylation in the biocontrol repertoire shown by these fungi is poorly understood.

### Trichostatin A (TSA) did not affect the overgrowth of *T*. *atroviride* on *R*. *solani*

In this work, we reported that TSA, a potent inhibitor of histone deacetylases class I and II, slightly affected the growth of *T*. *atroviride* and *R*. *solani*; however, the capability of *T*. *atroviride* to grow over the phytopathogen was not affected. Since TSA is *per se* an antifungal compound, it may have a negative effect on the growth of both fungi [48]. We also speculate that the inhibition of growth in both fungi could be due to the enhanced production of antimicrobial compounds promoted by the addition of TSA. In this regard, addition of TSA to the growing medium increases antimicrobial activity in a number of fungi against facultative bacterial and yeast pathogens [49]. Furthermore, *T*. *virens* strains that overproduce the antimicrobial compound gliovirin grow slower than the wt strain [49, 50]. Contrastingly, mutants lacking genes whose products are involved in the synthesis of antimicrobial compounds, such as the potent antimicrobial and cytotoxic compound gliotoxin grow faster than the wt strain [38, 51, 52].

### Addition of TSA to *T*. *atroviride*-growing medium enhanced its inhibitory effect on *R*. *solani* growth

Fungi are excellent producers of biological active compounds, as a consequence of their secondary metabolism, including antibiotics, plant growth-regulating molecules, cell toxic compounds, mutagenic, immunosuppressants, enzyme inhibitors, and molecules with other biological effects [25,53,54]. Here, we show that the addition of TSA enhanced the ability of *T*. *atroviride* MFCF to inhibit *R*. *solani* growth. These data suggest that inhibition of HDACs class I and II, such as Rpd3p and Hda1p, by TSA may be affecting the production of antimicrobial compounds in *T*. *atroviride*, probably by an increase in the acetylation of histone tails. This increased acetylation could de-repress silenced chromatin regions that contain genes involved in secondary metabolism and/or in the synthesis of antimicrobial compounds in this fungus. A genomic analysis of three *Trichoderma* species, including *T*. *atroviride*, showed that all of them contain the orthologous genes to *RPD3* and *HDA1* of *S*. *cerevisiae* [12]. It was recently demonstrated that the addition of HDAC inhibitors, including TSA, induces the antimicrobial activity of extracts on several fungi [49]. Moreover, *A*. *nidulans* mutants lacking components involved in chromatin compaction, such as HepA (heterochromatin protein 1) show de-repression of genes involved in the biosynthesis of secondary metabolites. In agreement with these results, the silent sterigmatocystin gene cluster shows low levels of repressive histone marks (trimethylated H3K9), whereas histone H3 acetylation is increased [55].

### TSA and *R*. *solani* presence negatively affected *T*. *atroviride ech-42*, *prb-1*, *pbs-1*, and *tps-1* gene expression

Expression analysis of mycoparasitism and secondary metabolism-related genes showed that *R*. *solani* induced the expression of *ech-42* and *prb-1*, as previously reported [28,56]. In addition, the presence of *R*. *solani* also induced the transcription of *tps-1* and the repression of *pbs-1*. A plausible explanation for the repression of *pbs-1* in the presence of *R*. *solani* is that the phytopathogen is trying to block the production of the peptaibols produced by *T*. *atroviride* through the synthesis of effector molecules. However, this is not the case for *tps-1*, which is involved in the synthesis of volatile terpenes. This fact, points to different regulation pathways for *pbs-1* and *ech-42*, *prb-1* and *tps-1* in the presence of the host. In this regard, it is well-known the strong battle that takes place between parasites and their hosts to counteract each other by means of lysing enzymes as well as effector molecules [57]. For instance, fungal effectors that promote pathogen virulence by suppressing the chitin-triggered immunity in plants, including LysM (effectors that carry no recognizable protein domains other than lysin motifs) have been identified also in saprophytic and beneficial fungi [58]. In this case, probably the phytopathogen is attempting to evade the mycophagous behavior of *T*. *atroviride* by producing effectors that, in the presence of TSA, enhance their production in the phytopathogen to silence genes such as *tps-1*.

Intriguingly, *pbs-1* and *ech-42*, *prb-1* and *tps-1* were positively affected by the presence of TSA. These results indicate that TSA may be affecting the transcription of these genes, by inhibiting Rpd3- and Hda1-encoding genes through histone acetylation in silenced chromatin regions as reported for other organisms [59,60]. In this sense, it is well known that TSA induces histone acetylation, which is often associated with an increased expression of a number of genes [61–63]. Frequently, these changes impact the biology of the organism, such as virulence and pathogenicity [64–66]. Moreover, the presence of both TSA and the phytopathogen downregulated the expression of all four genes, suggesting a positive effect of TSA in the synthesis of effector molecules in *R*. *solani* to counteract the mycoparasitic response of *T*. *atroviride*. Another plausible explanation is that both, the presence of TSA and the phytopathogen, but none of them alone enhances the expression of a negative regulator of mycoparasitism and secondary metabolism-related genes in this fungus.

### Deletion of *tgf-1* did not affect *T*. *atroviride* mycoparasitism effect against *R*. *solani*

Dual cultures of Δ*tgf-1* and *R*. *solani* showed that the mutant coiled around the phytopathogen hyphae; however, Δ*tgf-1* was deficient to grow over the pathogen. This result suggests that TGF-1 is not involved in coiling, but the reduced growth rate observed in the Δ*tgf-1* strain could be affecting its capacity to grow over *R*. *solani*. Probably, the *Trichoderma* growth rate is an important trait for competence. In this regard, it has been determined that growth inhibition of fungi by *Trichoderma* is directly proportional to the growth rate of the antagonists under a given condition [67].

### MFCF obtained from a Δ*tgf-1* strain shows enhanced inhibition of *R*. *solani* growth

Antimicrobial analysis of MFCF obtained from a Δ*tgf-1* strain, as well as dual cultures against *R*. *solani*, showed an enhanced growth inhibition of the phytopathogen compared to MFCF obtained from the wt strain. These results point out to a negative role of TGF-1 in secondary metabolism in *T*. *atroviride*. In this regard, TGF-1, orthologous in other organisms, also regulates negatively gene expression. For instance, in *N*. *crassa* and in *T*. *atroviride*, the orthologous to Gcn5 positively regulates gene photoinduction, but negatively the expression of the *al*-3 gene in darkness [5] (Uresti-Rivera et al., in preparation). In *S*. *cerevisiae*, stress response adaptation requires Gcn5 activity for the activation or repression of genes that are physically associated with this protein. This fact provides support for a role of Gcn5 and its orthologous proteins as co-activators as well as co-repressors [6].

Addition of TSA to the Δ*tgf-1* growing medium (PDB) reverted the effect of the lack of TGF-1 in the antibiosis assays, which suggests that histone acetylation induced by TSA is enough to totally revert the effect of *tgf-1* deletion, and that probably its product regulates negative elements. In this regard, it has been demonstrated that addition of TSA rescues the phenotypes of HAT mutants in yeast and HeLa cells [68–70]. Intriguingly, addition of TSA to the Vogel’s minimal medium, did not revert the effect of Δ*tgf-1* MFCF on *R*. *solani* to wild-type phenotype, on the contrary, it was enhanced. This result indicates that the components of the PDB medium could be affecting the acetylation pattern on the promoters of secondary metabolism-related genes in a Δ*tgf-1* background, but not in the wt.

### The Δ*tgf-1* strain shows constitutive chitinolytic but inductive proteolytic activity

Our results indicate that TGF-1 is a negative regulator of chitin degrading proteins, but not of those implicated in protein degradation, since chitinase activity showed an increased basal activity in the Δ*tgf-1* as compared to the wt. These results together with those of growth inhibition of *R*. *solani* may explain in part the augmented growth inhibition exerted by Δ*tgf-1* MFCF on the phytopathogen compared to wt MFCF, since some *Trichoderma* chitinases are secreted to the medium [71]. *Trichoderma* strains overexpressing either *ech-42* or *prb-1* provide more protection to plants against root and foliar pathogens [29–31]. In addition, mutants in the mitogen-activated protein kinase encoding gene, *tvk1*, from *T*. *virens*, showed increased transcription levels of mycoparasitism-related genes, high production of lytic enzymes and were considerably more effective in disease control than the wt strain [72].

### TGF-1 and TSA differentially regulate *ech-42*, *prb-1*, *pbs1*, and *tps-1* expression in the presence or absence of *R*. *solani*

Expression analysis of mycoparasitism- and secondary metabolism-related genes in the Δ*tgf-1* strain showed that probably the product of *tgf-1* negatively regulates *prb-1*, *tps-1*, *ech-42* and *pbs-1* in absence of the phytopathogen, since all tested genes were expressed in almost all tested times. Based on this, we can propose that TGF-1 could be a direct repressor through its putative histone acetyltransferase activity. Or it could be also an indirect repressor of these genes through acetylation of the promoter of a negative regulator-encoding gene, whose transcript is absent by the loss of TGF-1. In this regard, a negative role of Gcn5 has been observed in yeast and filamentous fungi in a number of processes [5, 6, 72] (Uresti-Rivera et al., in preparation).

Dual cultures of Δ*tgf-1* against *R*. *solani* showed that the expression of all tested genes in the mutant strain was downregulated at almost all tested times. A plausible explanation for these results is that TGF-1 is necessary to maintain the homeostasis with the effector molecules produced by *R*. *solani*, which in the mutant background favors the suppression of *Trichoderma* genes by the phytopathogen. Together, these results indicate that TGF-1 is necessary for the induction of all four genes in the presence of the phytopathogen or to counteract the negative effect of *R*. *solani* on *T*. *atroviride*. The exceptions in gene repression by *R*. *solani* at all tested times were *ech-42* and *pbs-1*. Probably, the induction of such genes under this condition may be due to the activity of other HATs such as Sas3 (ID Ta_223094; [12]), which has been shown to present overlapped roles with Gcn5 in *S*. *cerevisiae* [73]. Addition of TSA to the culture medium induced the expression of all tested genes in the Δ*tgf-1* strain, but at different times compared with its control. Probably this is a result of the promotion of histone acetylation, but it could be also a compensatory effect of other HAT in absence of TGF-1. The addition of TSA in the presence of *R*. *solani* induced the transcription of *ech-42* and *pbs-1*, which were repressed at 60 h of co-culture. Nevertheless, the expression of *prb-1* and *tps-1* genes was downregulated. A possible explanation for these results is that the sole presence of *R*. *solani* is not enough to fully repress the four genes at early times as it happened during confrontation with the wt strain in the presence of TSA and that such suppression was delayed by the absence of TGF-1.

The fact that other *Trichoderma* strains can grow over the Δ*tgf-1* mutant, and that some of them, including its parental strain, showed a marked lysing zone, could be explained as due to the slow growth of the Δ*tgf-1* strain. It is feasible that Δ*tgf-1* cannot compete with the other *Trichoderma* strains, although it showed an enhanced production of antibiotics that led to the lysis of other fungi hyphae [74], including its parental strain.

A detailed study focused on the interaction of HATs/HDACs with chromatin and acetylation balance, by ChIP on chip and ChIP-Seq assays, will be required to fully understand the role of chromatin modifications that regulate the antagonistic activity of *T*. *atroviride* and to determine if TGF-1, in these processes, acts as a co-activator as well as a co-repressor.

## Supporting information

S1 Fig Antibiosis assay using MFCF obtained from *T*. *atroviride* wt or Δ*tgf-1* strains grown in Vogel’s minimal medium in the presence or absence of TSA against *R*. *solani*. The Δ*tgf-1* and wt strains were grown for 7 days in Vogel’s minimal medium amended or not with 300 nM TSA, at 28 °C. MFCFs obtained from each of these cultures were added to PDA 1× medium at a final concentration of 60%. *R*. *solani* was inoculated into the different media and its radial growth was determined at 12, 24, 36, and 48 h. Radial growth of *R*. *solani* on PDA containing *T*. *atroviride* wt strain MFCF without TSA (black bars) or amended with TSA (crosshatched bars) was determined at the indicated times. Radial growth of *R*. *solani* on PDA containing *T*. *atroviride* Δ*tgf-1* strain MFCF without TSA (black dotted bars) or with TSA (arrow filled bars) was determined at the indicated times. The bars show the mean ± SD of three independent biological replicates. Different letters are used to indicate means that differ significantly (*P <* 0.05). Eight replicate plates were established for each treatment, and the experiment was repeated twice.
(TIF)

S1 Table Relation between growth inhibition of *R*. *solani* and the *T*. *atroviride* strain used in dual cultures assays. (DOCX)
