# Supplementary figures and images for: Histone acetyltransferase TGF-1 regulates Trichoderma atroviride secondary metabolism and mycoparasitism
Source: PLoS One. 2018 Apr 30;13(4):e0193872. doi: 10.1371/journal.pone.0193872 (PMC5927414; doi:10.1371/journal.pone.0193872)

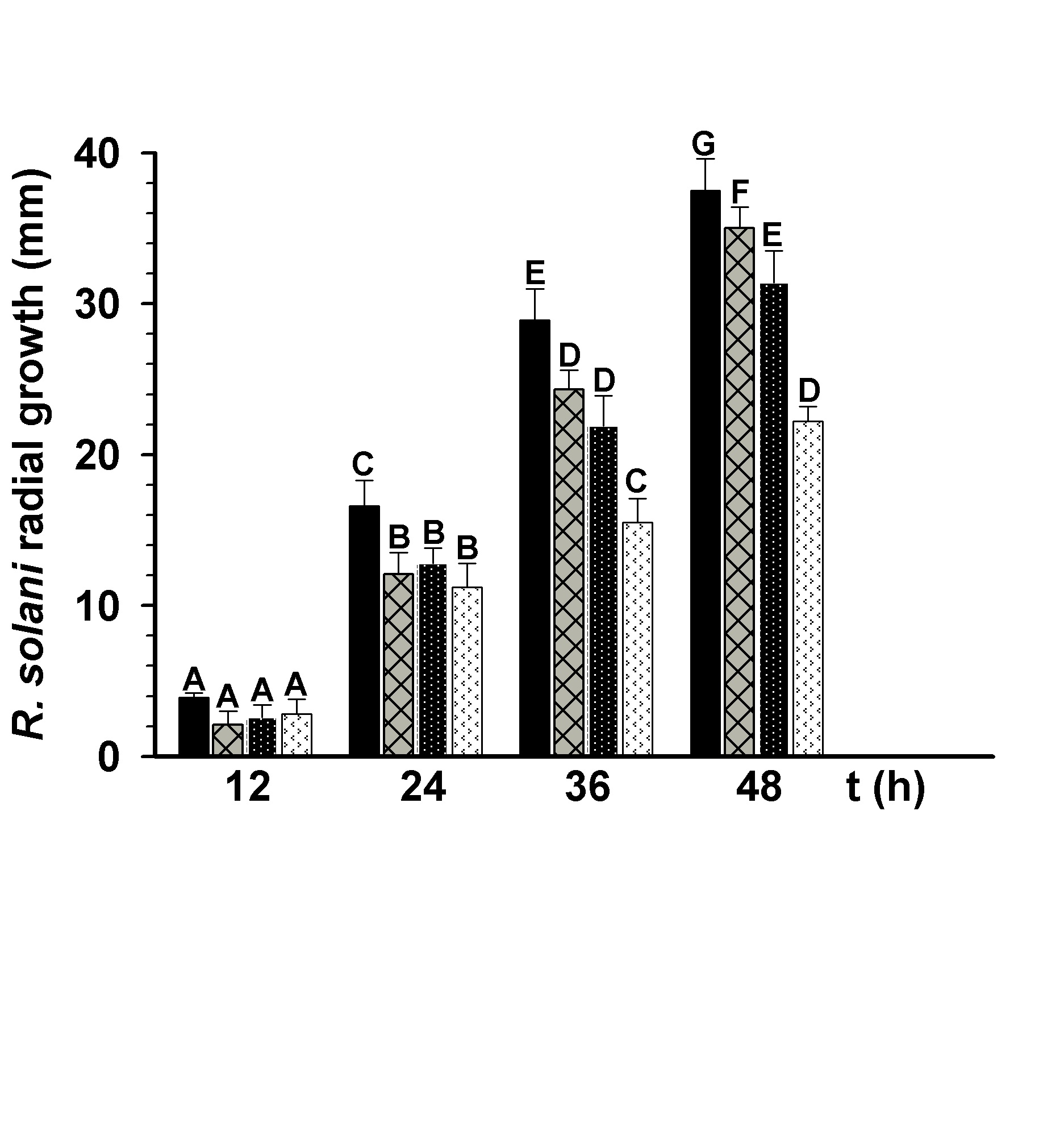

Supplement: S1 Fig — The Δtgf-1 and wt strains were grown for 7 days in Vogel’s minimal medium amended or not with 300 nM TSA, at 28 °C. MFCFs obtained from each of these cultures were added to PDA 1× medium at a final concentration of 60%. R. solani was inoculated into the different media and its radial growth was determined at 12, 24, 36, and 48 h. Radial growth of R. solani on PDA containing T. atroviride wt strain MFCF without TSA (black bars) or amended with TSA (crosshatched bars) was determined at the indicated times. Radial growth of R. solani on PDA containing T. atroviride Δtgf-1 strain MFCF without TSA (black dotted bars) or with TSA (arrow filled bars) was determined at the indicated times. The bars show the mean ± SD of three independent biological replicates. Different letters are used to indicate means that differ significantly (P < 0.05). Eight replicate plates were established for each treatment, and the experiment was repeated twice. (TIF) [file pone.0193872.s001.tif]
