# Supplementary material for: Histone acetyltransferase TGF-1 regulates Trichoderma atroviride secondary metabolism and mycoparasitism
Source: PLoS One. 2018 Apr 30;13(4):e0193872. doi: 10.1371/journal.pone.0193872 (PMC5927414; doi:10.1371/journal.pone.0193872)
Supplement: S1 Table — (DOCX) [file pone.0193872.s002.docx]

**S1 Table.** Relation between growth inhibition of *R. solani* and the *T. atroviride* strain used in dual cultures assays.

| Strain | Regression equation | Regression coefficient (R^2^) | Significance* |
| --- | --- | --- | --- |
| Control | Y = 1.421+0.959x | 0.930 | 0.000 |
| wt | Y = 1.731+0.414x | 0.802 | 0.000 |
| Δ*tgf-1* | Y = 1.616+0.056x | 0.382 | 0.014 |

x- denotes the parameters in the linear equation.

* Significant at 5%.
